# Risk of Post-stroke Epilepsy Following Stroke-Associated Acute Symptomatic Seizures

**DOI:** 10.3389/fnagi.2021.707732

**Published:** 2021-09-13

**Authors:** Ru Lin, Yaoyao Yu, Yi Wang, Emma Foster, Patrick Kwan, Mengqi Lin, Niange Xia, Huiqin Xu, Chenglong Xie, Yunjun Yang, Xinshi Wang

**Affiliations:** ^1^Department of Radiology, The First Affiliated Hospital of Wenzhou Medical University, Wenzhou, China; ^2^School of Public Health and Management, Wenzhou Medical University, Wenzhou, China; ^3^Department of Neuroscience, The Central Clinical School, Monash University, Melbourne, VIC, Australia; ^4^Department of Neurology, The First Affiliated Hospital of Wenzhou Medical University, Wenzhou, China; ^5^Key Laboratory of Alzheimer’s Disease of Zhejiang Province, Institute of Aging, Wenzhou Medical University, Wenzhou, China

**Keywords:** ischemic stroke, intracranial hemorrhage, symptomatic seizure, post-stroke epilepsy, predictors

## Abstract

**Objective:** Post-stroke epilepsy (PSE) is associated with increased morbidity and mortality. Stroke-associated acute symptomatic seizures are an important risk factor: 20.8–34.3% of these patients will go on to develop PSE. Identifying these “high risk” individuals may result in earlier PSE diagnosis, treatment, and avoidance of seizure-related morbidity. This study was to identify predictors of PSE development in patients with stroke-associated acute symptomatic seizures.

**Participants and Methods:** This was a retrospective cohort study of 167 patients with stroke-associated acute symptomatic seizures admitted to the Neurology Department of a tertiary Hospital of China, from 1 May 2006 to 30 January 2020. Both those with primary ischemic stroke and intracerebral hemorrhage were included in the study. Patient demographics, medical history, stroke-associated, and seizure-related variables were evaluated with univariable analysis and multivariable Cox regression analysis. PSE was defined as unprovoked seizures occurring > 7 days post-stroke. Data points were extracted from medical records and supplemented by tele-interview.

**Results:** Of the 167 patients with stroke-associated acute symptomatic seizures, 49 (29.3%) developed PSE. NIHSS score > 14 [hazard ratio (HR) 2.98, 95% CI 1.57–5.67], longer interval from stroke to acute symptomatic seizures (days 4–7 post-stroke) (HR 2.51, 95% CI 1.37–4.59) and multiple acute symptomatic seizures (HR 5.08, 95% CI 2.58–9.99) were independently associated with PSE development. This association remained in the sub-analysis within the ischemic stroke cohort. In the sub-analysis of the hemorrhagic stroke cohort, multilobar involvement (HR 4.80, 95% CI 1.49–15.39) was also independently associated with development of PSE. Further, we developed a nomogram to predict individual risk of developing PSE following stroke-associated acute symptomatic seizures. The nomogram showed a C-index of 0.73.

**Conclusion:** More severe neurofunctional deficits (NIHSS score > 14), longer interval from stroke to acute symptomatic seizures (days 4–7 post-stroke), and multiple acute symptomatic seizures were independently associated with development of PSE in patients with stroke-associated acute symptomatic seizures. This knowledge may increase clinical vigilance for development of PSE, facilitating rapid diagnosis and treatment initiation, and subsequently reduce seizure-related morbidity.

## Introduction

Stroke is the leading cause of epilepsy in the elderly, accounting for 30–50% of all-cause epilepsy in this group (Ruggles et al., 2001; Verellen and Cavazos, 2011; Zhao et al., 2018). Post-stroke seizures have been observed in 3.3–13.7% of patients with ischemic stroke (Lancman et al., 1993; Burn et al., 1997; Lamy et al., 2003; Guo et al., 2015; Koome et al., 2016; Chen et al., 2017, 2018; Stefanidou et al., 2017; Merkler et al., 2018; Naylor et al., 2018; Brondani et al., 2019) and 12.1–25% of patients with hemorrhagic stroke (Faught et al., 1989; Lancman et al., 1993; Burn et al., 1997; Madžar et al., 2014; Biffi et al., 2016; Claessens et al., 2017; Lahti et al., 2017; Merkler et al., 2018). The International League Against Epilepsy (ILAE) classifies seizures that occur within 7 days of strokes as acute symptomatic seizures (Beghi et al., 2010). These are thought to be provoked by acute disruption of brain integrity, dysfunction of metabolic homeostasis, and transient depolarizations of neurons (Camilo and Goldstein, 2004). Conversely, seizures occurring more than 7 days after strokes are defined as post-stroke epilepsy (PSE) since the risk of recurrent unprovoked seizures over the subsequent 10-year period is 71.5% (Hesdorffer et al., 2009) and thus fulfills ILAE criteria for epilepsy, i.e., post-stroke epilepsy (PSE) (Fisher et al., 2014). They are thought to be the long-term sequela of progressive structural changes secondary to the stroke, including neuronal loss, subsequent gliosis, and reorganization of neuronal networks (Sun et al., 2001, 2002; Camilo and Goldstein, 2004).

Previous studies have shown that stroke-associated acute symptomatic seizures do not significantly influence functional outcomes (De Herdt et al., 2011). In contrast, PSE can hamper post-stroke recovery and contribute to neurological deterioration (Kwan, 2010; Arntz et al., 2013b; Biffi et al., 2016). It is therefore important to identify those at increased risk of developing PSE. Several studies have identified stroke-associated acute symptomatic seizures as an important risk factor: 20.8–34.3% of these patients will go on to develop PSE (Roivainen et al., 2013; Haapaniemi et al., 2014; Lahti et al., 2017; Galovic et al., 2018), and these patients are also at increased risk of developing drug-resistant epilepsy (de Greef et al., 2015). However, most studies investigating risk factors associated with development of PSE were focusing on the entire cohort of patients with stroke, such as the SeLECT (Galovic et al., 2018) and CAVE score (Haapaniemi et al., 2014). Only a few studies focused on this higher-risk subgroup with acute symptomatic seizures that necessitate earlier initiation of anti-seizure medications. Glasgow Coma Scale (GCS) score ≤ 8 at presentation, larger hemorrhage volume, and larger intraventricular hemorrhage volume have been identified as predictors of PSE for patients with intracranial hemorrhage-associated acute symptomatic seizures (Biffi et al., 2016). A National Institute of Health stroke scale (NIHSS) score > 4 and post-stroke status epilepticus lasting > 16 h have been associated with a greater risk of developing PSE in those with stroke-associated status epilepticus (Abraira et al., 2019).

There remains several important limitations and knowledge gaps in this field. Existing studies are limited by sample size, with fewer than 100 patients included in each study. The factors associated with PSE development in those with ischemic stroke-associated acute symptomatic seizures are yet to be determined. Moreover, variables associated with the characteristics of acute symptomatic seizures were not included in these studies and therefore their correlation with development of PSE is unknown.

We aimed to identify the factors associated with PSE development in patients who have experienced acute symptomatic seizures following ischemic or hemorrhagic stroke, and to incorporate this into a nomogram for use at the bedside or outpatient clinic to predict PSE.

## Materials and Methods

### Subjects

This was a retrospective study of inpatients admitted to the Department of Neurology, the First Affiliated Hospital of Wenzhou Medical University, Wenzhou, China, from 1 May 2006 through 30 January 2020. The primary inclusion criterion was patients with a diagnosis of seizure, epilepsy, or status epilepticus, occurring within the initial 7-day period following a new-diagnosis stroke (i.e., patients with stroke-associated acute symptomatic seizures). Stroke types included primary ischemic stroke and primary intracranial hemorrhage as per ICD-10 codes. Exclusion criteria included: (1) patients without available computed tomography (CT) and/or magnetic resonance imaging (MRI); (2) patients with a prior diagnosis of epilepsy; (3) patients with secondary intracranial hemorrhage or venous infarction; (4) patients whose seizure might be attributed to other potential causes (brain tumor, intracranial vascular malformation, traumatic brain injury, etc.); and (5) patients who were lost to follow-up or died within 3 months of the stroke incident; (6) patients who reported ambiguous event occurring > 7 days post-stroke. This study was approved by the Ethics Committee of our hospital (No.2020-185). Verbal informed consent was obtained from the subject or the subject’s legally authorized representative through tele-interview.

### Recognition of Seizures and Patient Follow-Up

Acute symptomatic seizures were defined as seizures occurring within 7 days of a stroke as per the ILAE definition (Beghi et al., 2010). PSE was diagnosed in patients with unprovoked seizures occurring 7 days after a stroke in the context of stroke-associated epileptogenic substrate as noted on neuroimaging. Presence of PSE seizures was ascertained by consensus agreement from two epileptologists (XW and NX), with disagreements adjudicated by a senior epileptologist (HX). Patients that reported ambiguous seizure-like events were conservatively excluded from the analysis. Other information collected included use of anti-seizure medications (ASMs), development of stroke-associated neurological symptoms (e.g., hemiparesis), and death. These were extracted from medical records, and supplemented by tele-interview using a modified standardized questionnaire (Table 1; Keezer et al., 2014). All participants were followed up until seizure recurrence, occurrence of a further stroke, death, or end of the study period (May 30, 2020).

**TABLE 1:** Post-stroke epilepsy screening questionnaire.

**Q1: Self-reported diagnosis**
a. Has ever been diagnosed by a doctor to have seizures/epilepsies/convulsions after discharge? ^a^
b. How soon after discharge did you experience the first recurrent seizure? ^b^
Only patients responded Q1a with “yes” were recorded as post-stroke epilepsy, if not, patients were asked by following questions.
**Q2: Symptom-based screening questions: a, b**
a. Have you ever had, or has anyone told you that you had any of the following symptom after discharge? ^c^
i. A seizure, convulsive, fit or spell under any circumstances?
ii. Uncontrolled movement of part or all of your body such as twitching, jerking, shaking, or going limp?
iii. An unexplained change in your mental state or level or unawareness; or an episode of “spacing out” that you could not control?
iv. Shortly after waking up, either in the morning or after a nap, have you ever noticed uncontrollable jerking or clumsiness, such as dropping things or things suddenly “flying” from your hands?
v. Have you ever had repeated unusual spells?
b. How soon after discharge did the symptom happen? ^b^
**Q3: Questions about Anti-seizure medications?**
a: Are you currently using any anti-seizure drug including “valproate,” “levetiracetam,” “carbamazepine,” or “oxcarbazepine”?
b: If not, when did you stop the drug? ^d^
a Acceptable answers to each of the questions include: “yes,” “no,” “possible,” or “don’t know.”
b Only if the patient or caregiver can provide the approximate date or we can obtain it from the medical records, he/she were included in analysis.
c Patients were diagnosed with PSE only if they fulfill 1 + 2 + 3 or 1 + 4 of the following: 1. “yes” for Q2a-i or ii, 2. Provide an approximate date for Q2b, 3. “yes” for Q3a, 4. confirmed by medical records. If they respond “no” for any of the questions, they were recorded as non-PSE. Otherwise, they’re excluded from analysis to ensure the best accuracy of diagnosis.
d An approximate year

*PSE, post-stroke epilepsy.*

### Variables

#### Stroke-Associated Variables

Stroke was categorized into primary ischemic stroke and intracranial hemorrhage. Ischemic stroke was further classified according to the TOAST (Trial of Org 10172 in Acute Stroke Treatment) criteria, i.e., large-artery atherosclerosis, cardioembolism, small-vessel occlusion, stroke of other determined etiology, and stroke of undetermined etiology (Adams et al., 1993). Stroke severity at admission was recorded as per the NIHSS. Medical treatment of stroke was categorized as thrombolysis, lipid-lowering therapy, antiplatelet therapy, anticoagulation, as well as surgical interventions. Variables related to stroke localization included cortical involvement, multilobar involvement, deep region involvement, as well as ventricular extension of hematoma. Volumetric assessment of the lesion location was completed through three-dimensional reconstruction of the regions of interest and manual depiction of the lesion circumference in multiple successive layers (Zhang et al., 2020). Infectious complications that occurred during hospitalization, such as respiratory tract infections, were also recorded.

#### Other Variables

Other variables collected included demographic information, i.e., sex; age at time of stroke; concomitant medical diseases, i.e., hypertension, diabetes, hyperlipidemia, and hyperuricemia; and lifestyle factors, i.e., current smoking and alcohol intake status.

#### Seizure-Related Variables

Acute symptomatic seizure-related variables included time interval (days) from stroke to seizure; presence of multiple acute symptomatic seizures, defined as ≥ 2 seizures occurring over ≥ 24 h time period but within 7 days of the stroke; and presence of status epilepticus, as per the ILAE diagnostic criteria (Trinka et al., 2015). Only events with clear focal motor or focal to bilateral tonic-clonic semiology were included in the study. Given the retrospective nature of the study, potential non-motor seizure events were conservatively excluded to reduce the risk of erroneously including ambiguous, non-seizure events. Electroencephalogram (EEG) results within 1 week of acute symptomatic seizure onset were classified as either normal, non-specific abnormalities, and/or epileptiform discharges. ASM regimen at the time of most recent follow-up was recorded. The time interval (days) from the stroke to first unprovoked seizure was recorded.

### Statistical Analysis

The primary endpoint was time to first unprovoked seizure. Age and stroke lesion volume were analyzed both as continuous and categorical variables, specifically, age < 65 years and lesion volume > 10 ml, as these factors have previously been associated with intracranial hemorrhage-associated PSE (Haapaniemi et al., 2014). Other factors were analyzed as categorical variables. Cut-off points with the best sensitivity and specificity to predict PSE development were identified for the NIHSS score and the time interval from stroke to acute symptomatic seizure. Univariable analysis were conducted using the Kaplan-Meier method with a log-rank test or univariable Cox regression. Variables with *p* ≤ 0.1 in the univariable analysis were then selected into the multivariable Cox proportional hazard regression analysis using “survival” package with a forward stepwise method based on likelihood ratio, to establish the fitted model and calculate the hazard ratio (HR). Censored patients in the Kaplan-Meier analysis and Cox regression analysis included those who experienced a further stroke before experiencing their first unprovoked seizure; those who were lost to follow up; those who died due to non-seizure related causes; and those who did not experience unprovoked seizures within the study period. A nomogram based on the multivariable analysis of the total cohort was built using the “rms” package, and Harrell’s C-index was measured to quantify the discrimination performance of the nomogram. Forest plot displaying the results of multivariable analysis was completed with the “forest” package. Two-tailed *p*-values < 0.05 were considered to be statistically significant. Statistical analysis was conducted with R software (version 4.0.3).

## Results

**FIGURE 1:**
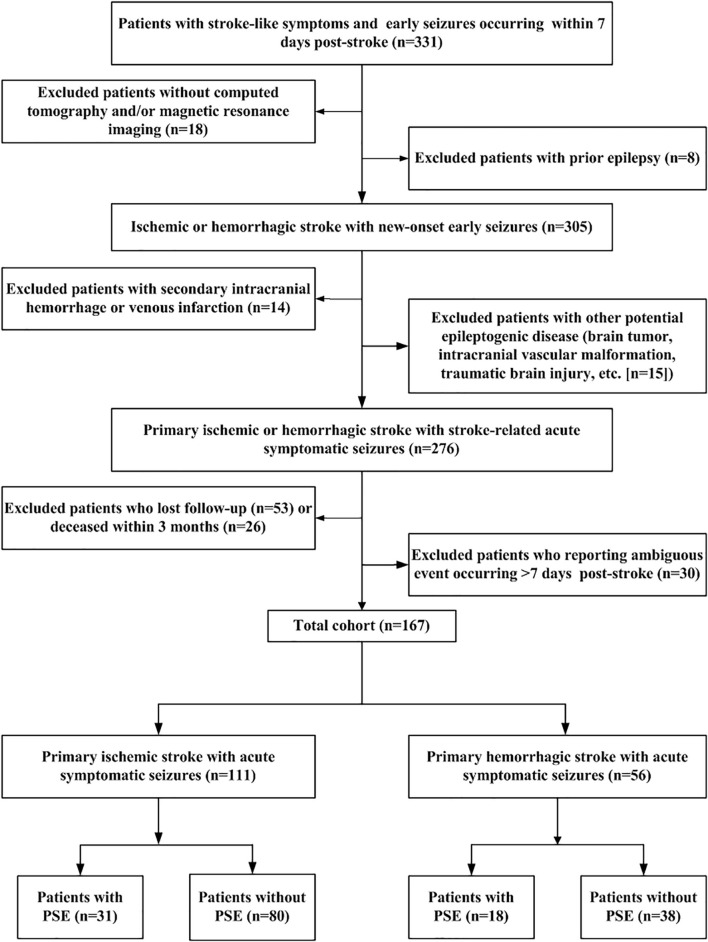
Flow chart of patient recruitment. Flow-chart summarizing sequential application of inclusion/exclusion criteria leading to definition of final study cohort. PSE: post-stroke epilepsy.

A total of 331 patients met the primary inclusion criteria with a stroke and early seizures occurring within the initial 7-day period. Among them, 167 met all eligibility criteria for new-onset acute symptomatic seizure secondary to the index stroke and were retained for analyses (Figure 1).

### Characteristics of All Subjects With Stroke-Associated Acute Symptomatic Seizures

**FIGURE 2:**
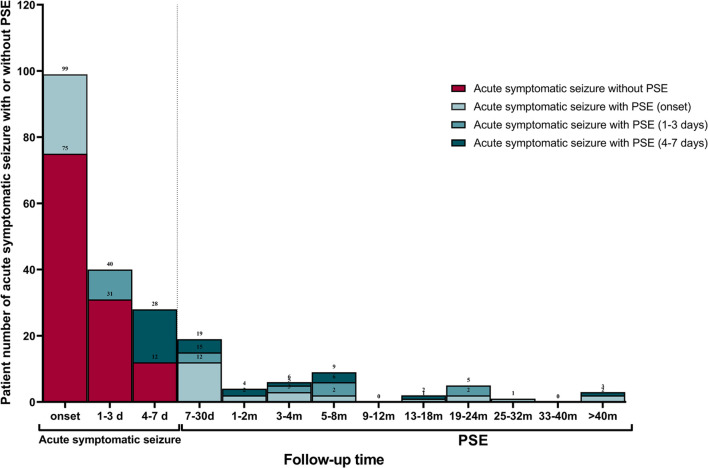
Late recurrent seizure incidence over time. Frequency and distribution of patients with early seizures occurred within 7 days of index stroke and PSE occurred after 7 days of index stroke. PSE, post-stroke epilepsy.

Table 2 displays characteristics of the study cohort. The final study cohort comprised of 111 (66.5%, *n* = 111/167) patients with primary ischemic stroke and 56 (33.5%, *n* = 56/167) with primary intracranial hemorrhage. Approximately half of these patients (54.5%, *n* = 91/167) experienced bilateral tonic-clonic seizures and 66 (39.5%, *n* = 66/167) experienced focal motor seizures. The median time of acute symptomatic seizure onset was 0 days [interquartile range (IQR) 0–2] post-stroke. Only a small proportion of patients (16.8%, *n* = 28/167) experienced acute symptomatic seizures > 3 days post-stroke. Status epilepticus was recorded in 44 patients (26.3%, *n* = 44/167). Multiple acute symptomatic seizures occurred in 21 patients (12.6%, *n* = 21/167). Only 38.3% (*n* = 64/167) of patients underwent EEG recording during their acute inpatient hospitalization. Of these, 26 (40.6%) had epileptiform discharges, 35 (54.7%) had non-specific abnormalities, and 3 (4.7%) were normal. Most of the patients (80.2%, *n* = 134/167) received ASM therapy for their acute symptomatic seizures. Twenty-nine (59.2%) patients were still taking ASM therapy at the time they experienced their first PSE-related unprovoked seizure, and 23 (19.5%) patients were still on ASM therapy at the end of the study without having experienced an unprovoked seizure. The median follow-up time was 592 days (IQR 170–1512). PSE developed in 49 (29.3%) patients: 18 (32.1%, *n* = 18/56) of those with hemorrhagic stroke, and 31 (27.9%, *n* = 31/111) of those with ischemic stroke. The PSE incidence over time is displayed in Figure 2.

**TABLE 2:** Baseline characteristics of the total cohort.

Variables	*n* = 167
**Demographics**	
Male sex, No./total No. (%) Female sex, n(%)	114/167 (68.3)
Age at onset (years), mean (SD)	64.4 (12.9)
**Medical history**	
Alcohol drinking, No./total No. (%)	68/167 (40.7)
Smoking, No./total No. (%)	73/167 (43.7)
Hypertension, No./total No. (%)	121/167 (72.5)
Diabetes, No./total No. (%)	38/167 (22.8)
Hyperlipidemia, No./total No. (%)	12/167 (7.2)
Hyperuricemia, No./total No. (%)	45/167 (26.9)
**Characteristics of stroke**	
Ischemic stroke, No./total No. (%)	111/167 (66.5)
NIHSS score > 14, No./total No. (%)	31/167 (18.6)
Location of the stroke, No./total No. (%)^a^	
Cortical involvement	142/167 (85.0)
Multilobar involvement	83/167 (49.7)
Deep region involvement	61/167 (36.5)
Lesion volume (ml), median (IQR)	9.0 (2.7–24.0)
Infectious complication, No./total No. (%)	58/167 (34.7)
Follow-up duration (days), median (IQR)	592 (170–1512)
**Characteristics of seizures**	
Seizure type, No./total No. (%)	
Bilateral tonic-clonic seizure	91/167 (54.5)
Focal motor seizure	66/167 (39.5)
Undetermined	10/167 (6.0)
Stroke-to-acute symptomatic seizure interval (days), median (IQR)	0 (0–2)
Stroke-to-acute symptomatic seizure interval > 3 days, No./total No. (%)	28/167 (16.7)
Multiple acute symptomatic seizures, No./total No. (%)	21/167 (12.6)
Status epilepticus, No./total No. (%)	44/167 (26.3)
EEG, No./total No. (%)	
Normal	3/167 (1.8)
Non-specific abnormality	35/167 (21.0)
Epileptiform discharge	26/167 (15.6)
Absent	103/167 (61.7)
ASM treatment, No./total No. (%)	134/167 (80.2)
PSE, No./total No. (%)	49/167 (29.3)

*Continuous variables with normal distribution were presented as mean [standard deviation (SD)]; non-normal variables were presented as median [interquartile range (IQR)]; quantitative variables were presented as number/total number (%). NIHSS, National Institute of Health stroke scale; ASM, anti-seizure medication; EEG, electroencephalogram; PSE, post-stroke epilepsy.*

*^a^ Some patients were counted in more than one group.*

### Analysis of PSE Predictors in the Total Cohort

**FIGURE 3:**
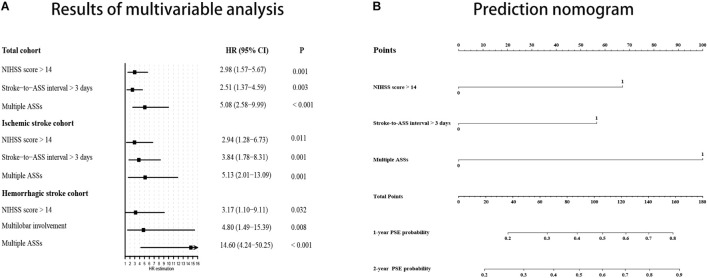
PSE probability prediction using results of multivariable analysis. **(A)** Forest plot based on results of multivariable analysis. **(B)** Prediction nomogram on the basis of results multivariable analysis in the total cohort. NIHSS, National Institute of Health stroke scale; ASS, acute symptomatic seizure; PSE, post-stroke epilepsy; 95% CI, 95% confidence interval.

The results of univariable analyses are displayed in Table 3. NIHSS score > 14 at admission and longer interval from stroke to acute symptomatic seizures (days 4–7 post-stroke) were significantly more likely in those who went on to develop PSE compared to those who did not (32.7% vs. 12.7%, *p* = 0.002 and 32.7% vs. 10.2%, *p* < 0.001, respectively). Multiple acute symptomatic seizures were also significantly associated with subsequent development of PSE (26.5% vs. 6.8%, *p* < 0.001). There were no statistical differences for other variables between the PSE and non-PSE subgroups. In the Cox proportional hazard model, NIHSS score > 14 at admission (HR 2.98, 95% CI 1.57–5.67, *p* = 0.001), multiple acute symptomatic seizures (HR, 5.08; 95% CI, 2.58–9.99; *p* < 0.001), and longer interval from stroke to acute symptomatic seizures (days 4–7 post-stroke) (HR 2.51, 95% CI 1.37–4.59, *p* = 0.003) were associated with PSE following stroke-associated acute symptomatic seizure(s) (Figure 3A).

**TABLE 3:** Univariable analysis of baseline characteristics associated with development of PSE.

Variables	Total cohort			Ischemic stroke cohort			Hemorrhagic stroke cohort		
	PSE (*n* = 49)	Non-PSE (*n* = 118)	*P*-value	PSE (*n* = 31)	Non-PSE (*n* = 80)	*P*-value	PSE (*n* = 18)	Non-PSE (*n* = 38)	*P*-value
**Demographics**									
Male sex, No./total No. (%)	33/49 (67.3)	81/118 (68.6)	0.86	19/31 (61.3)	55/80 (68.8)	0.65	14/18 (77.8)	26/38 (68.4)	0.38
Age at onset (years), mean (SD)	64.5 (11.2)	64.3 (13.6)	0.50	67.8 (8.9)	65.5 (12.1)	0.34	59.8 (13.0)	61.6 (16.2)	0.91
Age < 65 years, No./total No. (%)	23/49 (46.9)	59/118 (50.0)	0.47	11/31 (35.5)	37/80 (46.3)	0.31	12/18 (66.7)	22/38 (57.8)	0.97
**Medical history**									
Alcohol drinking, No./total No. (%)	19/49 (38.8)	49/118 (41.5)	0.79	11/31 (35.5)	30/80 (37.5)	0.93	8/18 (44.4)	19/38 (50.0)	0.66
Smoking, No./total No. (%)	19/49 (38.8)	54/118 (45.8)	0.42	12/31 (38.7)	37/80 (46.3)	0.70	7/18 (38.9)	17/38 (44.7)	0.36
Hypertension, No./total No. (%)	37/49 (75.5)	85/118 (72.0)	0.56	20/31 (64.5)	52/80 (65.0)	0.86	17/18 (94.4)	32/38 (84.2)	0.18
Diabetes, No./total No. (%)	12/49 (24.5)	26/118 (22.0)	0.64	8/31 (25.8)	20/80 (25.0)	0.95	4/18 (22.2)	6/38 (15.8)	0.54
Hyperlipidemia, No./total No. (%)	4/49 (8.2)	8/118 (6.8)	0.60	2/31 (6.5)	3/80 (3.8)	0.31	2/18 (11.1)	5/38 (13.2)	0.90
Hyperuricemia, No./total No. (%)	12/49 (24.5)	33/118 (28.0)	0.88	8/31 (25.8)	22/80 (27.5)	0.98	4/18 (22.2)	11/38 (28.9)	0.77
**Characteristics of seizures**									
Seizure type, No./total No. (%)			0.43			0.94			0.10
Bilateral tonic-clonic seizure	26/49 (53.1)	65/108 (60.2)		17/31 (54.8)	41/75 (54.7)		9/18 (50.0)	24/33 (72.7)	
Focal motor seizure	23/49 (46.9)	43/108 (39.8)		14/31 (45.2)	34/75 (45.3)		9/18 (50.0)	9/33 (23.3)	
Stroke-to-acute symptomatic seizure interval (days), median (IQR)	0.0 (0.0–4.0)	0.0 (0.0–2.0)	0.01	0.0 (0.0–4.0)	0.0 (0.0–1.0)	0.03	2.0 (0.0–4.0)	1.0 (0.0–2.0)	0.23
Stroke-to- acute symptomatic seizure interval > 3 days, No./total No. (%)	16/49 (32.7)	12/118 (10.2)	<0.001	11/31 (35.5)	8/80 (10.0)	0.002	5/18 (27.8)	4/38 (10.5)	0.08
Multiple acute symptomatic seizures, No./total No. (%)	13/49 (26.5)	8/118 (6.8)	<0.001	7/31 (22.6)	7/80 (8.8)	0.01	6/18 (33.3)	1/38 (2.6)	< 0.001
Status epilepticus, No./total No. (%)	16/49 (32.7)	28/118 (23.7)	0.36	10/31 (32.3)	18/80 (22.5)	0.50	6/18 (33.3)	10/38 (26.3)	0.59
EEG, No./total No. (%)			0.27			0.85			0.15
Normal	0/19 (0.0)	3/45 (6.7)		0/12 (0.0)	1/37 (2.7)		0/7 (0.0)	2/8 (25.0)	
Non-specific abnormality	9/19 (47.4)	26/45 (57.8)		7/12 (58.3)	23/37 (62.1)		2/7 (28.6)	3/8 (37.5)	
Epileptiform discharge	10/19 (52.6)	16/45 (35.6)		5/12 (41.7)	13/37 (35.1)		5/7 (71.4)	3/8 (37.5)	
ASM treatment, No./total No. (%)	41/49 (83.7)	93/118 (78.8)	0.44	28/31 (90.3)	61/80 (76.3)	0.09	13/18 (72.2)	32/38 (84.2)	0.23
**Characteristics of stroke**									
NIHSS score > 14, No./total No. (%)	16/49 (32.7)	15/118 (12.7)	0.002	9/31 (29.0)	10/80 (12.5)	0.04	7/18 (38.9)	5/38 (13.2)	0.02
Infectious complication, No./total No. (%)	19/49 (38.8)	39/118 (33.1)	0.39	12/31 (38.7)	25/80 (31.3)	0.52	7/18 (38.9)	14/38 (36.8)	0.66
Lesion volume (ml), median (IQR)	9.8 (2.3–28.6)	9.0 (3.1–21.9)	0.86	9.0 (1.1–21.6)	7.6 (1.9–25.9)	0.53	14.2 (5.0–42.5)	13.4 (4.9–21.0)	0.18
Lesion volume > 10 ml, No./total No. (%)	23/49 (46.9)	51/118 (43.2)	0.65	14/31 (45.2)	30/80 (37.5)	0.50	9/18 (50.0)	18/38 (47.4)	0.72
Location of stroke, No./total No. (%)^a^									
Cortical involvement	41/49 (83.7)	101/118 (85.6)	0.69	24/31 (77.4)	75/80 (93.8)	0.02	17/18 (94.4)	25/38 (65.8)	0.04
Multilobar involvement	27/49 (55.1)	58/118 (49.2)	0.35	20/31 (64.5)	50/80 (62.5)	0.61	7/18 (38.9)	6/38 (15.8)	0.08
Deep region involvement	16/49 (32.7)	45/118 (38.1)	0.64	12/31 (38.7)	33/80 (41.3)	0.89	2/18 (11.1)	11/38 (28.9)	0.65
Extension into ventricle, No./total No. (%)							3/18 (16.7)	8/38 (23.7)	0.85
Surgical treatment, No./total No. (%)							4/18 (22.2)	5/38 (13.2)	0.29
TOAST classification, No./total No. (%)						0.33			
Large-artery atherosclerosis				22/31 (71.0)	43/80 (53.8)				
Cardioembolism				2/31 (6.5)	11/80 (13.8)				
Small-vessel occlusion				3/31 (9.7)	17/80 (21.3)				
Stroke of other determined etiology				2/31 (6.5)	5/80 (6.3)				
Stroke of undetermined etiology				2/31 (6.5)	4/80 (5.0)				
Thrombolysis, No./total No. (%)				1/31 (3.2)	3/80 (3.8)	1.00			
Lipid-lowering therapy, No./total No. (%)				25/31 (80.6)	68/80 (85.0)	0.69			
Thrombocytopenic therapy, No./total No. (%)				28/31 (90.3)	60/80 (75.0)	0.10			
Anticoagulation, No./total No. (%)				8/31 (25.8)	19/80 (23.8)	0.80			
**Follow-up duration (days), median (IQR)**	74 (17–201)	993 (496–2056)		55 (15–202)	963 (504–1,669)		126 (30–242)	1,155 (472–2,622)	

*Continuous variables with normal distribution were presented as mean [standard deviation (SD)]; non-normal variables were presented as median [interquartile range (IQR)]; quantitative variables were presented as number/total number (%). PSE, post-stroke epilepsy; NIHSS, National Institute of Health stroke scale; ASM, anti-seizure medication; EEG, electroencephalogram.*

*^a^ Some patients were counted in more than one group.*

### Analysis of PSE Predictors Within the Ischemic Stroke Cohort

A subgroup analysis was performed to evaluate predictors of PSE in those with ischemic stroke-associated acute symptomatic seizures. NIHSS score > 14 (29.0% vs. 12.5%, *p* = 0.04), multiple acute symptomatic seizures (22.6% vs. 8.8%, *p* = 0.01), and longer interval from stroke to acute symptomatic seizures (days 4–7 post-stroke) (35.5% vs. 10.0%, *p* = 0.002) were associated with development of PSE in the univariable analysis. Interestingly, cortical involvement of the stroke territory (77.4% vs. 93.8%, *p* = 0.004) was less common in patients that went on to develop PSE compared to those without PSE, and reached statistical significance in the univariable analysis (Table 2). In the subsequent Cox regression analysis, NIHSS score > 14 (HR 2.94, 95% CI 1.28–6.73, *p* = 0.01), acute symptomatic seizures occurring > 3 days post-stroke (HR 3.84, 95% CI 1.77–8.31, *p* = 0.001), and multiple acute symptomatic seizures (HR 5.13, 95% CI 2.01–13.09, *p* = 0.001) were associated with PSE development (Figure 3A).

### Analysis of PSE Predictors Within the Hemorrhagic Stroke Cohort

We also conducted sub-analyses for patients with hemorrhagic stroke-associated acute symptomatic seizures. Significant predictors included NIHSS scores > 14 (38.9% vs. 13.2%, *p* = 0.02), multiple acute symptomatic seizures (33.3% vs. 2.6%, *p* < 0.001), and stroke lesions with cortical involvement (94.4% vs. 65.8%, *p* = 0.04). In the Cox proportional hazard model, multiple acute symptomatic seizures (HR 14.60, 95% CI 4.24–50.25, *p* < 0.001), NIHSS score > 14 at admission (HR 3.17, 95% CI 1.10–9.11, *p* = 0.03) and multilobar involvement (HR 4.80, 95% CI 1.49–15.39, *p* = 0.008) were associated with PSE development following a hemorrhagic stroke-associated acute symptomatic seizure (Figure 3A).

### Development of a Clinical Prediction Nomogram

We built a nomogram based on the Cox proportional hazard model in the total cohort to predict an individual’s risk of developing PSE following stroke-associated acute symptomatic seizures (Figure 3B). In the nomogram, an interval from stroke to acute symptomatic seizures > 3 days (days 4–7 post-stroke) equals 57.5 points, multiple acute symptomatic seizures equal 100 points, a NIHSS score > 14 equals 67.5 points. The corresponding risk of PSE was < 20%, approximately 27, 31, 47, 60, and > 80% for a patient with total points of 0, 57.5, 67.5, 100, 125 (57.5 + 67.5), and ≥ 160, respectively, during 1-year follow-up; and it was about 20, 35, 39, 58, 72, and ≥ 90% during 2-year follow-up. The C-index for the prediction nomogram was 0.73.

## Discussion

This study revealed that people with stroke-associated acute symptomatic seizures are more likely to develop PSE if they have more severe neurofunctional deficits at time of stroke presentation; if their initial acute symptomatic seizure occurs between days 4 and 7 post-stroke; if the stroke involves multilobar and if patients experience multiple acute symptomatic seizures within the first week post-stroke. These factors have been incorporated into a nomogram to assist clinicians in calculating the risk of PSE development for individual patients, which in turn may assist with management decisions.

Our study found that seizures occurring between days 4 and 7 post-stroke were associated with a higher risk of developing PSE, compared to early seizures that occurred between days 0 and 3 post-stroke. This finding is consistent with previous studies which reported that acute symptomatic seizures mainly occurred within 3 days of stroke onset (Haapaniemi et al., 2014), and seizures that occurred later were associated with higher risk of long-term seizure recurrence (Sung and Chu, 1990; Bladin et al., 2000). These findings suggest that the definition of stroke-associated acute symptomatic seizures may be revised to those occurring within 3 days of the stroke onset, as opposed to the current definition of occurring within 7 days. The occurrence of multiple seizures within the first week following a stroke was a predictor for future PSE. Although these multiple early seizures all fell within the current definition of an “acute symptomatic seizure” timeframe, they may in fact represent the early stages of PSE. Supporting this, microarray analysis in a rat ischemic model has found that down-regulation of several ion channels and receptors begins as early as 1 day post-stroke (Lu et al., 2004).

Consistent with previous studies, our work found that stroke severity is strongly associated with development of PSE (Arntz et al., 2013a; Graham et al., 2013; Jungehulsing et al., 2013; Zhang et al., 2014). A previous study identified GCS score ≤ 8 at time of stroke admission was an important risk factor for development of PSE in those with intracranial hemorrhage-associated acute symptomatic seizures (Biffi et al., 2016). Although the GCS was not designed for this purpose, it may be interpreted as a surrogate marker for stroke severity. A previous study examined factors associated with development of PSE for patients with stroke-associated status epilepticus. An NIHSS cut score of > 4 at time of stroke admission was found to be a significant predictor of future development of PSE (Abraira et al., 2019). Our study found the significant NIHSS cut-score to be > 14, indicating more extensive neuronal damage was required to cross the PSE threshold for our patients. The lower NIHSS threshold for patients with status epilepticus may be due to the additional risk that status epilepticus carries in terms of developing PSE (Tomari et al., 2017).

Large stroke lesion volume has previously been associated with development of PSE, particularly in patients with hemorrhagic strokes (Bladin et al., 2000; Haapaniemi et al., 2014). However, this association was not found in our study. Instead, we found multilobar involvement to be an important predictor for the development of PSE. This finding has also been noted in other studies (Lancman et al., 1993; Beghi et al., 2011). Although large stroke volume and multilobar involvement represent similar measures, the latter may also be interpreted as a surrogate marker for more severe neurofunctional deficits. Supporting this, we found the lesion volume was significantly larger and NIHSS score was significantly higher in patients with multilobar involvement compared to those with single lobe involvement (Table 4).

**TABLE 4:** Comparing NHISS score and lesion volume between patients with and without multilobar involvement in hemorrhagic stroke cohort.

	With multilobar involvement (*n* = 16)	Without multilobar involvement (*n* = 40)	*P*-value
NIHSS score, median (IQR)	11 (6–17)	4 (2–12)	0.015
Lesion volume (ml), median (IQR)	18.9 (10.0–45.5)	9.0 (3.9–21.4)	0.023

*IQR, interquartile range; NIHSS, National Institute of Health stroke scale.*

There is conflicting evidence regarding the association between cortical involvement of stroke and risk of development of PSE. In the univariable analysis, those with hemorrhagic stroke-associated acute symptomatic seizures were more likely to develop PSE if there was cortical involvement compared to if there was not; this has also been reported previously (Haapaniemi et al., 2014; Biffi et al., 2016; Galovic et al., 2018). Conversely, cortical involvement was negatively associated with PSE development in those with ischemic stroke-associated acute symptomatic seizures; previous studies have reported similarly (Tomari et al., 2017; Abraira et al., 2019). Certainly, cortical involvement is a well-recognized risk factor for stroke-associated acute symptomatic seizures. The resultant cortical irritation may directly damage cortical neurons, causing increased neuronal excitability and leading to the onset of seizures in the acute stroke phase (Arboix et al., 1997; Roivainen et al., 2013). The negative association between cortical involvement and development of PSE is not necessarily indicative of a “protective” effect of cortical involvement. Rather, the development of acute symptomatic seizures in those without direct cortical irritation may be indicative of an increased predisposition to epileptic seizures. This may be genetic or lesional. For example, strokes within the deep brain matter may occur in individuals with small-vessel disease, which in turn is associated with development of epilepsy in the elderly (Pitkanen et al., 2016). Further research is needed to confirm this hypothesis.

Our study has important practical implications in terms of identifying those at higher risk for developing PSE following stroke-associated acute symptomatic seizures. This group may benefit from closer clinical follow-up, which may facilitate earlier PSE diagnosis and treatment initiation. This in turn may reduce the risk of future seizure-related morbidity and mortality. Our study reveals several important directions for future research. These include investigating the temporal association of stroke-associated acute symptomatic seizures and development of PSE by observing dynamic changes of critical molecules; exploring the shared pathophysiological mechanisms of neuronal damage and development of epileptic seizures induced by stroke; and eliciting the mechanisms underlying the predisposition for developing PSE. Further, our findings may be used to target high risk patients for recruitment in clinical trials that evaluate interventions to prevent PSE.

For example, based on the nomogram, for a patient whose seizures occur between days 4–7 post-stroke, if he/she has no other risk factors, the total points would be 57.5 and the risk of PSE is only 27% following the first year post-stroke and 34% following the second year; but if he/she has also a NIHSS score > 14, the total points would be 125 and the risk of PSE is about 60% following the first year post-stroke and 72% following the second year; if he/she experiences multiple seizures within 7 days post-stroke, he/she will get additional 100 points and thus the risk of PSE will be very high (>80%). Not only closer clinical follow-up should be conducted for these patients, but also prophylactic anti-seizure treatment might be necessary in view of the high PSE risk for the latter two conditions, especially the last one.

Our study has several limitations. As with all studies with a medical chart review component, identifying patients with acute symptomatic seizures relied on comprehensive medical documentation. Although it was felt that motor seizures could be correctly identified with reasonable confidence by both the treating clinician and the researchers who subsequently reviewed the medical chart, there was less certainty around the diagnosis for patients with subtle, non-motor events. As such, the latter events were excluded from the study. This focus on specificity rather than sensitivity resulted in a highly curated dataset, but potentially at the expense of under-reporting acute symptomatic seizure events. The follow-up diagnosis of PSE rested largely on patient and caregiver reports. PSE-seizures may present with non-motor semiology, and so may not be obvious to non-medically trained people. Therefore, it is possible that the true incidence of PSE was also under-reported.

In addition, a high proportion of males (68%) were included in our cohort as compared to other studies (Biffi et al., 2016; Abraira et al., 2019). As noted, an important difference between previous studies and our study is that we only included motor seizures. Therefore, a possible explanation is that women are more prone to non-motor seizures post-stroke. A hospital-based study has found that the female percentage of the patients with post-stroke non-convulsive status epilepticus (NCSE) was higher than that of the stroke patients without NCSE (50% vs. 35.9%) (Belcastro et al., 2014). Although the difference did not reach statistical significance, this might be due to the small sample size as there were only 32 patients with post-stroke NCSE (Belcastro et al., 2014). Further investigations were needed to verify the hypothesis. On the other hand, the male proportion is comparable to that of some large-sized stroke registry cohorts in China with 60–67.5% males (Wei et al., 2010; Zhang et al., 2021). This might be ascribed to the well-documented higher incidence of stroke in men than in women in all age classes (Reeves et al., 2008) and the higher male proportion in Chinese population.

## Conclusion

In summary, patients with an NIHSS score > 14, acute symptomatic seizures occurring > 3 days post-stroke, and multiple acute symptomatic seizures were at higher risk of developing PSE compared to patients with stroke-associated acute symptomatic seizures without these factors. In addition, multilobar involvement was also a predictor of PSE in patients with primary hemorrhagic stroke-associated acute symptomatic seizures.

## Data Availability Statement

The raw data supporting the conclusions of this article will be made available by the authors, without undue reservation.

## Ethics Statement

The studies involving human participants were reviewed and approved by the Review of Ethics Committee in Clinical Research of the First Affiliated Hospital of Wenzhou Medical University. The ethics committee waived the requirement of written informed consent for participation.

## Author Contributions

XW and YYa designed this study, obtained funding for this study, and supervised this study. ML and XW drafted this manuscript. XW, PK, EF, and YYa made critical revision of this manuscript for important intellectual content. ML, YYu, and YW conducted statistical analysis of this study. XW, HX, and YYa gave administrative, technical, or material supports. All authors collected the data.

## Conflict of Interest

The authors declare that the research was conducted in the absence of any commercial or financial relationships that could be construed as a potential conflict of interest.

## Publisher’s Note

All claims expressed in this article are solely those of the authors and do not necessarily represent those of their affiliated organizations, or those of the publisher, the editors and the reviewers. Any product that may be evaluated in this article, or claim that may be made by its manufacturer, is not guaranteed or endorsed by the publisher.
